# Attitude Estimation of Underwater Vehicles Using Field Measurements and Bias Compensation

**DOI:** 10.3390/s19020330

**Published:** 2019-01-15

**Authors:** Nak Yong Ko, Seokki Jeong, Suk-seung Hwang, Jae-Young Pyun

**Affiliations:** 1 Department of Electronic Engineering, Chosun University, 375 Seosuk-dong Dong-gu, Gwangju 501-759, Korea; hwangss@chosun.ac.kr; 2 Department of Control and Instrumentation Engineering, Graduate School, Chosun University, 375 Seosuk-dong Dong-gu, Gwangju 501-759, Korea; seak-ki@hanmail.net; 3 Department of Information and Communication Engineering, Chosun University, 375 Seosuk-dong Dong-gu, Gwangju 501-759, Korea; jypyun@chosun.ac.kr

**Keywords:** field measurement, gravitational field, magnetic field, attitude estimation, underwater vehicles, Kalman filter, Euler angles

## Abstract

This paper proposes a method of estimating the attitude of an underwater vehicle. The proposed method uses two field measurements, namely, a gravitational field and a magnetic field represented in terms of vectors in three-dimensional space. In many existing methods that convert the measured field vectors into Euler angles, the yaw accuracy is affected by the uncertainty of the gravitational measurement and by the uncertainty of the magnetic field measurement. Additionally, previous methods have used the magnetic field measurement under the assumption that the magnetic field has only a horizontal component. The proposed method utilizes all field measurement components as they are, without converting them into Euler angles. The bias in the measured magnetic field vector is estimated and compensated to take full advantage of all measured field vector components. Because the proposed method deals with the measured field independently, uncertainties in the measured vectors affect the attitude estimation separately without adding up. The proposed method was tested by conducting navigation experiments with an unmanned underwater vehicle inside test tanks. The results were compared with those obtained by other methods, wherein the Euler angles converted from the measured field vectors were used as measurements.

## 1. Introduction

Navigation is one of the fundamental technologies for underwater vehicles and robots. The attitude and velocity of a vehicle is required for the navigation and control of the vehicle [1,2]. This paper proposes a method that improves the accuracy of attitude estimation for which measurements sensed by the Microelectromechanical Systems-Attitude and Heading Reference System (MEMS-AHRS) are used. The MEMS-AHRS measures the acceleration, magnetic field, and angular rate of the sensor in three directions. The measured acceleration is regarded as the measurement of the gravitational field, and the measured magnetic field is regarded as the measurement of the geomagnetic field specific to a geographic location. The MEMS-AHRS has a small volume and is an inexpensive, lightweight, and convenient sensor for determining attitude. In this paper, MEMS-AHRS will be referred to simply as attitude and heading reference system (AHRS).

Amongst the three measurements by an AHRS, the magnetic field measurement is highly susceptible to distortion, owing to the hard-iron and soft-iron effects. Additionally, the scale factor error and non-orthogonality of the sensor construction make the measurement less reliable. The uncertainty in the magnetic field measurement degrades the attitude determined by the quaternion estimator (QUEST) [3,4]. In the QUEST algorithm, the magnetic field measurement affects not only the yaw estimation, but also the estimation of roll and pitch. To reduce the effect of the magnetic field measurement on the estimation of roll and pitch, a method that restricts the use of magnetic field measurement only to the determination of rotation about the vertical axis has been proposed [4].

The calibration of a magnetometer measurement is one of the major research areas for improving the attitude estimation based on AHRS measurement. A batch linear least squares method, which determines the bias, scale factor error, and non-orthogonality, has been proposed for real-time calibration [5]. Another method has applied Kalman filters (KFs) to the magnetic field measurement and gravity vector, respectively, to enhance the reliability of field measurements. The outputs from these KFs were fed to an extended Kalman filter (EKF) for attitude estimation [6].

Many AHRS based methods use partial field measurement information, and the inaccuracy of roll and pitch deteriorates the yaw calculation [4,7]. Additionally, the yaw estimate is less reliable than the estimate of the roll and pitch because the yaw estimate depends on the magnetic field measurement, which is regarded as the measurement of geomagnetic field. The magnetic field measurement of the AHRS is vulnerable to magnetic interference induced by the vehicle and its surrounding environment. Although it is desirable to detect only the geomagnetic field, the hard iron and soft iron effects add a magnetic field other than the geomagnetic field and also distort the geomagnetic field [2].

Various attitude estimation methods convert the acceleration and magnetic field measurements into Euler angles, namely, the roll, pitch, and yaw angles. The converted Euler angles are used in the measurement update stage of the estimation procedure [7]. The methods calculate the roll and pitch from the vertical component of the acceleration measurement, and convert the magnetic field into yaw, under the assumption that the horizontal component of the magnetic field points north with some declination from true north. Because the calculation of yaw requires roll and pitch, which are converted from acceleration, the uncertainty in the acceleration measurement propagates to the yaw error through the roll and pitch error.

Many of the underwater navigation methods are based on Bayesian estimation approaches. The EKF is one of the prevalent Bayesian estimation approaches toward estimating attitude. The EKF predicts the attitude using an angular rate measurement and corrects the predicted attitude using measurements. The measurements are the attitude calculated from the acceleration and magnetic field measurements. The roll and pitch are calculated from the measured acceleration under the assumption that the measured acceleration is attributed only to gravity. The yaw is calculated using the calculated roll and pitch and the magnetic field measurement. Therefore, the accuracy of yaw is affected by the accuracy of the roll, pitch, and magnetic field measurement [4]. In addition to the interference in the magnetic field measurement, the uncertainty of the calculated roll and pitch deteriorates the calculation of yaw [7].

The unscented Kalman filter (UKF) and particle filter (PF) are also the Bayesian approaches for underwater navigation. As one of the variants of KF, UKF provides improved estimation accuracy while keeping the computation load comparable with the EKF [8]. The feasibility of UKF is verified by offline implementation using triangular navigation trajectory. The method utilized propulsion system modelling, and the estimation results of the UKF are compared with those of EKF, where an ultra-short base line (USBL) system provides ground truth for comparison. Another UKF based method incorporated a dynamic model of the vehicle as well as the kinematic model into the process model of the estimator. This method is tested through offline implementation using open-sea test data [9]. Finally, the UKF is used for real-time estimation and the estimated results are used for control of the underwater vehicle [10].

The PF, which is a sample based implementation of the Bayesian estimation approach, is also used for underwater navigation. The PFs used landmarks or features of underwater environments such as seabed terrain information [11] and gravity vector [12] specific for locations in the underwater environments. PF is also used for localization of acoustic sensors in underwater environments [13]. In these applications, PF outperforms other methods in dealing with highly nonlinear and complicated measurement model where explicit mathematical description of the environment is impractical.

The complementary filter (CF) method fuses the complementary spectral characteristics of the field measurements and angular rate measurement of the AHRS for attitude estimation [14,15]. Although the angular rate measurement yields an attitude change that is accurate and useful for short-term use, the attitude calculated from the change exhibits drift with time. Thus, its long-term reliability is compromised. On the contrary, the roll and pitch calculated from the acceleration and magnetic field measurement provide the absolute attitude, which is robust in the long term without drift. However, the roll and pitch are not suitable for determining the relative change of attitude in the short-term. Based on this complementary property, the attitude calculated from the angular rate is passed through a high-pass filter, while the attitude calculated from the acceleration and magnetic field is passed through a low-pass filter. Then, the filtered attitudes are combined by the CF, which is being further developed to adjust the gains based on the measurement of acceleration for high acceleration applications [16]. The nonlinear explicit complementary filter (NECF) is partially similar to the CF and has been widely used with many modifications [2,17].

The NECF was derived as an observer in a special orthogonal group SO(3) by exploiting the geometry of the SO(3) [15,17]. It uses the difference between the detected field measurements and the presumably true field through the cross product of the measured field and the true field pair. The method estimates the rotation matrix by reflecting the attitude error on the bias in the angular rate, while the bias in the measured field is not explicitly addressed. The NECF uses only the horizontal component of the magnetic field measurement. In the context of an invariant observer [18], it has been demonstrated that a particular choice of gains reduces the invariant observer to NECF [19].

The problem of representing the attitude and rotation has been investigated extensively because the attitude and rotation constitute SO(3), which is not a linear space. The methods of invariant EKF (IEKF) systematically resolve the nonlinear system estimation problem by using a transformation group based on Lie algebra [19,20,21]. The application of IEKF depends on the measurements available to the specific problem. Therefore, it is still hard to apply IEKF to a general attitude estimation problem, where the AHRS measurements and the additional field measurements are utilized.

The sine rotation vector (SRV) method has been proposed to relieve the problem arising from the Euler angle representation of measurement innovation [22]. As a measurement innovation for the KF application, the SRV method replaces the algebraic difference between the measured Euler angles and the predicted Euler angles with the sine rotation vector. Although the SRV method improves the estimation performance, it still requires the conversion of field measurements into Euler angles, and also requires additional computation to calculate the SRV. The EKF, CF, and SRV basically use the Euler angles converted from the acceleration and magnetic field measurement. Therefore, they all suffer from a distorted magnetic field measurement and uncertainty in the yaw calculation.

This paper describes a method that overcomes the problem of distortion-prone magnetic field measurement and accumulated error in yaw calculation. The proposed method fully utilizes the field measurements and calculates yaw independently from the roll and pitch by using all components of the measured acceleration and magnetic field directly in the measurement update stage without converting them to Euler angles. Additionally, the proposed method estimates and compensates for the magnetic field bias, and uses the magnetic field vector to estimate the roll and pitch as well as the yaw. The proposed method uses the well-established EKF method, which is easily expandable when additional field measurements other than gravity and magnetic field are available. For example, if a landmark is recognized, then the vector directional to the landmark is regarded as the measured field. Thus, a simple augmentation of the measurement variables and measurement model equations is carried out by appending the additional field, and this completes the EKF adaptation to this particular problem.

Section 2 formulates the problem in a manner suitable to the use of field measurements. Section 3 explains the proposed approach in the order of algorithm flow: prediction, bias estimation and compensation, application of measurement model to field measurement, and correction. This section also discusses an optional alternative, which can be used when the vertical component of the magnetic field bias is poorly compensated and the incorrect compensation degrades the estimation performance. Section 4 describes the experiments using unmanned underwater vehicles (UUVs) navigating in test tanks. The results compare the attitude estimation performance and trajectory estimation performance for which the attitude estimated by the proposed method is used. Finally, Section 5 concludes this paper along with suggestions for future work related to the proposed method.

## 2. Problem Formulation

### 2.1. Nomenclature

The notations used for the derivation of the method are as follows:

x(t)attitude of a robot at time *t*; $x\left(t\right)={\left(\phi (t),\theta (t),\psi (t)\right)}^{T}$ where ϕ(t), θ(t), and ψ(t)
indicate the roll, pitch, and yaw, respectively${\hat{x}}^{-}\left(t\right)$attitude predicted for time *t*, before being corrected using the measurements;
${\hat{x}}^{-}\left(t\right)={\left({\hat{\phi }}^{-}\left(t\right),{\hat{\theta }}^{-}\left(t\right),{\hat{\psi }}^{-}\left(t\right)\right)}^{T}$$\hat{x}\left(t\right)$attitude estimated for time *t* through the prediction and correction procedure;
$\hat{x}\left(t\right)={\left(\hat{\phi }\left(t\right),\hat{\theta }\left(t\right),\hat{\psi }\left(t\right)\right)}^{T}$a(t)acceleration measured at time *t* in the instrument coordinate frame;
$a\left(t\right)={\left(a_{x}\left(t\right),a_{y}\left(t\right),a_{z}\left(t\right)\right)}^{T}$$a^{u}\left(t\right)$normalized acceleration measurement; $a^{u}\left(t\right)={\left(a_{x}^{u}\left(t\right),a_{y}^{u}\left(t\right),a_{z}^{u}\left(t\right)\right)}^{T}=\frac{a(t)}{∥a(t)∥}$$\tilde{m}\left(t\right)$magnetic field measured in the instrument coordinate frame;
$\tilde{m}\left(t\right)={\left({\tilde{m}}_{x}\left(t\right),{\tilde{m}}_{y}\left(t\right),{\tilde{m}}_{z}\left(t\right)\right)}^{T}$$b_{m}\left(t\right)$bias in the magnetic field measurement $\tilde{m}\left(t\right)$; $b_{m}\left(t\right)={\left(b_{m_{x}}\left(t\right),b_{m_{y}}\left(t\right),b_{m_{z}}\left(t\right)\right)}^{T}$m(t)magnetic field wherein the bias $b_{m}\left(t\right)$ is compensated from measurement $\tilde{m}\left(t\right)$;
$m\left(t\right)={\left(m_{x}\left(t\right),m_{y}\left(t\right),m_{z}\left(t\right)\right)}^{T}=\tilde{m}\left(t\right)-b_{m}\left(t\right)$$m^{u}\left(t\right)$normalized magnetic field measurement;
$m^{u}\left(t\right)={\left(m_{x}^{u}\left(t\right),m_{y}^{u}\left(t\right),m_{z}^{u}\left(t\right)\right)}^{T}=\frac{m(t)}{∥m(t)∥}$$m_{g}\left(t\right)$geomagnetic field represented in the North-East-Down (NED) coordinate frame
appropriate for the geographical location; $m_{g}\left(t\right)={\left(m_{g,x}\left(t\right),m_{g,y}\left(t\right),m_{g,z}\left(t\right)\right)}^{T}$$z^{u}\left(t\right)$normalized field measurement at time *t*; $z^{u}\left(t\right)={\left(a^{u}\left(t\right),m^{u}\left(t\right)\right)}^{T}$u(t)linear velocity measured by a Doppler velocity log (DVL) in the instrument coordinate
frame; $u\left(t\right)={\left(u(t),v(t),w(t)\right)}^{T}$ω(t)rotational (angular) velocity measured by a gyroscope in the instrument coordinate frame;
$\omega \left(t\right)={\left(p\left(t\right),q\left(t\right),r\left(t\right)\right)}^{T}$f(·)motion function relating attitude x(t) and angular velocity ω(t) in the instrument
coordinate frame to the robot’s angular velocity $\dot{x}\left(t\right)$; $\dot{x}\left(t\right)=f\left(x(t),\omega (t)\right)$h(·)measurement function relating state x(t) to measurement $z^{u}\left(t\right)$; $z^{u}\left(t\right)=h\left(x\left(t\right)\right)$$t_{k}$the *k*th discretized sampling instant of time$\Delta t_{k}$time period between $t=t_{k-1}$ and $t=t_{k}$; $\Delta t_{k}=t_{k}-t_{k-1}$


In many studies, an underwater robot or vehicle is often referred to as a UUV. Thus, in the following sections, the terms UUV, vehicle, and robot will be used interchangeably.

### 2.2. Problem Formulation

The problem being considered in this paper is described as follows.

**Attitude Estimation Problem:** Find the attitude $\hat{x}\left(t\right)$ of an underwater robot at time $t=t_{k}$, given the measurements $a(t_{k})$ and $\tilde{m}\left(t_{k}\right)$, and attitude $\hat{x}\left(t_{k-1}\right)$ estimated at time $t=t_{k-1}$, linear velocity $u(t_{k})$, and angular velocity $\omega (t_{k})$.

The state x(t) to be estimated consists of the Euler angles of a robot; namely, the roll ϕ(t), pitch θ(t), and yaw ψ(t).

(1) $$x\left(t\right)={\left(\phi (t),\theta (t),\psi (t)\right)}^{T}.$$

The acceleration measurement $a\left(t\right)={\left(a_{x}\left(t\right),a_{y}\left(t\right),a_{z}\left(t\right)\right)}^{T}$ is normalized to $a^{u}\left(t\right)={\left(a_{x}^{u}\left(t\right),a_{y}^{u}\left(t\right),a_{z}^{u}\left(t\right)\right)}^{T}$. The magnetic field measurement $\tilde{m}\left(t\right)={\left({\tilde{m}}_{x}\left(t\right),{\tilde{m}}_{y}\left(t\right),{\tilde{m}}_{z}\left(t\right)\right)}^{T}$ is calibrated to m(t) by subtracting the bias from the measurement. Then, the bias calibrated magnetic field measurement m(t) is normalized to $m^{u}\left(t\right)={\left(m_{x}^{u}\left(t\right),m_{y}^{u}\left(t\right),m_{z}^{u}\left(t\right)\right)}^{T}$.

The normalized measurements $a^{u}\left(t\right)$ and $m^{u}\left(t\right)$ are merged to form the measurement vector z(t), as expressed by Equation (2):

(2) $$z\left(t\right)=z^{u}\left(t\right)={\left(a_{x}^{u}\left(t\right),a_{y}^{u}\left(t\right),a_{z}^{u}\left(t\right),m_{x}^{u}\left(t\right),m_{y}^{u}\left(t\right),m_{z}^{u}\left(t\right)\right)}^{T}.$$

The field vectors a(t) and $\tilde{m}\left(t\right)$ are measured in the instrument coordinate frame. It is assumed that the vehicle coordinate frame coincides with the instrument coordinate frame. In the development, the reference coordinate frame, which is otherwise called the world coordinate frame, indicates the North–East–Down (NED) coordinate frame [23].

A vehicle’s linear velocity $u\left(t\right)={\left(u(t),v(t),w(t)\right)}^{T}$ is detected by a DVL, and the detected velocity is used to dead-reckon the vehicle location that will be used to verify the applicability and performance of the proposed attitude estimation method. The rotational velocity of the vehicle $\omega \left(t\right)={\left(p\left(t\right),q\left(t\right),r\left(t\right)\right)}^{T}$ is detected by a gyroscope to provide the time update of the state. The linear velocity u(t) and rotational velocity ω(t) are also measured in the vehicle coordinate frame, which is adjusted to coincide with the instrument coordinate frame.

The procedure for attitude estimation consists of prediction, bias estimation, bias compensation, and correction, and will be described in the following sections.

## 3. Attitude Estimation

The attitude estimation process consists of the prediction and correction of the attitude and attitude covariance. The process uses a magnetic field measurement, whose bias is estimated and compensated. This section explains the prediction stage (Section 3.1), procedure for the estimation and compensation of the magnetic field bias (Section 3.2), derivation of measurement model (Section 3.3), and correction stage (Section 3.4). Additionally, an optional method using only the horizontal component of the magnetic field is applicable when the severe distortion of the vertical component of the magnetic field measurement degrades the attitude estimation. This method is presented in Section 3.5.

### 3.1. Prediction of State and State Covariance

The time update, which is otherwise called the prediction or propagation step, predicts the state for time $t_{k}$ as ${\hat{x}}^{-}\left(t_{k}\right)$ and the covariance $P^{-}\left(t_{k}\right)$ of the predicted state ${\hat{x}}^{-}\left(t_{k}\right)$. The prediction step updates the previous estimates $\hat{x}\left(t_{k-1}\right)$ and the covariance $P(t_{k-1})$ by using the differential equation for attitude transition. The differential equation model of the attitude is expressed by the following Equation (3):

(3) $$\left(\begin{array}{c} \dot{\phi }\left(t\right)\\ \dot{\theta }\left(t\right)\\ \dot{\psi }\left(t\right) \end{array}\right)=\underset{f(x(t),\omega (t))}{\underset{︸}{\left(\begin{array}{c} p(t)+q(t)S(\phi (t))T(\theta (t))+r(t)C(\phi (t))T(\theta (t))\\ q(t)C(\phi (t))-r(t)S(\phi (t))\\ q(t)S(\phi (t))\sec(\theta (t))+r(t)C(\phi (t))\sec(\theta (t)) \end{array}\right)}}+\underset{v(t)}{\underset{︸}{\left(\begin{array}{c} v_{\phi }\left(t\right)\\ v_{\theta }\left(t\right)\\ v_{\psi }\left(t\right) \end{array}\right)}},$$

(4) $$\dot{x}\left(t\right)=f\left(x\left(t\right),\omega \left(t\right)\right)+v\left(t\right),\;v\left(t\right)∼N\left(0,Q\left(t\right)\right).$$

The time derivative $\dot{x}\left(t\right)$ of the state in Equation (4) is a function of the measured angular velocity $\omega \left(t\right)={\left(p\left(t\right),q\left(t\right),r\left(t\right)\right)}^{T}$ and state $x\left(t\right)={\left(\phi \left(t\right),\theta \left(t\right),\psi \left(t\right)\right)}^{T}$. In these equations, S(·), C(·) and T(·) represent sin(·), cos(·), and tan(·), respectively. In this model, v(t), which is assumed to be an additive and Gaussian with zero mean, is the noisy uncertainty involved in the state transition.

The prediction of the state is based on the derivative of $\hat{x}\left(t\right)$ as expressed by Equation (5):

(5) $$\dot{\hat{x}}\left(t\right)=f\left(\hat{x}\left(t\right),\omega \left(t\right)\right).$$

In Equation (6), the derivative $\dot{P}\left(t\right)$ is used to predict the state covariance P(t):

(6) $$\dot{P}\left(t\right)=F\left(t\right)P\left(t\right)+P\left(t\right)F^{T}\left(t\right)+Q\left(t\right),$$

(7) $$\mathrm{where},\;\;F\left(t\right)\equiv {\left.\frac{\partial f(x(t),\omega (t))}{\partial x(t)}\right|}_{x\left(t\right)=\hat{x}\left(t\right)}.$$

The Jacobian matrix F(t) is expressed by Equation (8):

(8) $$F\left(t\right)\equiv {\left.\frac{\partial f(x(t),\omega (t))}{\partial x(t)}\right|}_{x\left(t\right)=\hat{x}\left(t\right)}=\left(\begin{array}{ccc} \left(qC\left(\hat{\phi }\right)-rS\left(\hat{\phi }\right)\right)T\left(\hat{\theta }\right) & \left(qS\left(\hat{\phi }\right)+rC\left(\hat{\phi }\right)\right)\sec^{2}\left(\hat{\theta }\right) & 0\\ -qS\left(\hat{\phi }\right)-rC\left(\hat{\phi }\right) & 0 & 0\\ \left(qC\left(\hat{\phi }\right)-rS\left(\hat{\phi }\right)\right)\sec\left(\hat{\theta }\right) & \left(qS\left(\hat{\phi }\right)+rC\left(\hat{\phi }\right)\right)\sec\left(\hat{\theta }\right)T\left(\hat{\theta }\right) & 0 \end{array}\right),$$

where the time variable (t) is deleted from p(t), q(t), r(t), $\hat{\phi }\left(t\right)$, and $\hat{\theta }\left(t\right)$, for notational simplicity.

Using Equations (5) and (6), the state and covariance are predicted for time $t_{k}$ from state $\hat{x}\left(t_{k-1}\right)$ and covariance $P(t_{k-1})$, which are estimated at time $t_{k-1}$. The predicted state ${\hat{x}}^{-}\left(t_{k}\right)$ and covariance $P^{-}\left(t_{k}\right)$ can be calculated by the numerical integration of (5) and (6) for time $t\in [t_{k-1},t_{k})$. One of the simplest methods to predict them is to use Equations (9) and (10):

(9) $${\hat{x}}^{-}\left(t_{k}\right)=\hat{x}\left(t_{k-1}\right)+\dot{\hat{x}}\left(t_{k-1}\right)\left(t_{k}-t_{k-1}\right),$$

(10) $$P^{-}\left(t_{k}\right)=P\left(t_{k-1}\right)+\dot{P}\left(t_{k-1}\right)\left(t_{k}-t_{k-1}\right).$$

This method can be used under the provision that $\Delta t_{k}=t_{k}-t_{k-1}$ is small enough for derivatives $\dot{\hat{x}}\left(t_{k-1}\right)$ and $\dot{P}\left(t_{k-1}\right)$ to approximate the derivatives $\dot{\hat{x}}\left(t\right)$ and $\dot{P}\left(t\right)$, respectively, for the time period $t\in [t_{k-1},t_{k})$.

### 3.2. Bias Estimation and Compensation of Magnetic Field Measurement

After the prediction procedure described in Section 3.1, the predicted attitude and covariance will be corrected by comparing the measurements with the predicted measurements which are calculated based on the predicted state values. However, the magnetic field measurement is corrupted by noise and bias. Therefore, before applying the measurement update procedure, the bias changing with time and the location must be estimated and compensated for. The proposed method estimates the bias by a Kalman filter (KF) using the angular rate measurement [24].

The measurement of the magnetic field $\tilde{m}\left(t\right)$ includes bias $b_{m}\left(t\right)$ and Gaussian noise $n_{m}\left(t\right)$, as modeled in Equation (11):

(11) $$\tilde{m}\left(t\right)={}_{r}^{v}R\left(t\right){\;}^{r}m_{tr}\left(t\right)+b_{m}\left(t\right)+n_{m}\left(t\right),\;n_{m}\left(t\right)∼N\left(0,Q_{m}\left(t\right)\right),$$

where ${}^{r}m_{tr}\left(t\right)$ is the true magnetic field in the reference coordinate frame, while $\tilde{m}\left(t\right)$ represents the measured magnetic field in the instrument coordinate frame. Ignoring the Gaussian noise $n_{m}\left(t\right)$ results in the true magnetic field ${}^{r}m_{tr}\left(t\right)$ in the fixed world coordinate frame of Equation (12):

(12) $${}^{r}m_{tr}\left(t\right)={{}_{r}^{v}R}^{-1}\left(t\right)\left(\tilde{m}\left(t\right)-b_{m}\left(t\right)\right)={}_{v}^{r}R\left(t\right)\left(\tilde{m}\left(t\right)-b_{m}\left(t\right)\right).$$

In Equation (12), ${}_{v}^{r}R\left(t\right)$ is the rotation matrix that transforms a vector in the vehicle coordinate frame to the vector in the fixed world coordinate frame, expressed by Equation (13):

(13) $$\begin{array}{ll} {}_{v}^{r}R\left(t\right) & =\left(\begin{array}{ccc} C(\theta )C(\psi ) & S(\phi )S(\theta )C(\psi )-C(\phi )S(\psi ) & C(\phi )S(\theta )C(\psi )+S(\phi )S(\psi )\\ C(\theta )S(\psi ) & S(\phi )S(\theta )S(\psi )+C(\phi )C(\psi ) & C(\phi )S(\theta )S(\psi )-S(\phi )C(\psi )\\ -S(\theta ) & S(\phi )C(\theta ) & C(\phi )C(\theta ) \end{array}\right)\\ & =\left(\begin{array}{ccc} {}_{v}^{r}R_{x}\left(t\right) & {}_{v}^{r}R_{y}\left(t\right) & {}_{v}^{r}R_{z}\left(t\right) \end{array}\right). \end{array}$$

Differentiating Equation (12) results in Equation (14):

(14) $$0={}_{v}^{r}\dot{R}\left(t\right)\left(\tilde{m}\left(t\right)-b_{m}\left(t\right)\right)+{}_{v}^{r}R\left(t\right)\dot{\tilde{m}}\left(t\right).$$

When deriving Equation (14), it is assumed that the true magnetic field at a location does not change rapidly. Thus, there is no substantial change in the magnetic field with time. Additionally, the same assumption applies to the bias $b_{m}\left(t\right)$. Applying the relationship ${}_{v}^{r}\dot{R}\left(t\right)={}_{v}^{r}R\left(t\right)S\left(\omega \left(t\right)\right)$ to Equation (14) gives the following equation:

(15) $${}_{v}^{r}R\left(t\right)\dot{\tilde{m}}\left(t\right)=-{}_{v}^{r}R\left(t\right)S\left(\omega \left(t\right)\right)\left(\tilde{m}\left(t\right)-b_{m}\left(t\right)\right),$$

where the skew-symmetric cross product matrix S(ω(t)) is defined as Equation (16):

(16) $$S\left(\omega \left(t\right)\right)=\left(\begin{array}{ccc} 0 & -\omega_{z} & \omega_{y}\\ \omega_{z} & 0 & -\omega_{x}\\ -\omega_{y} & \omega_{x} & 0 \end{array}\right).$$

Multiplying ${}_{v}^{r}R^{-1}\left(t\right)$ on both sides of Equation (15) yields the differential equation of the magnetic field measurement $\tilde{m}\left(t\right)$:

(17) $$\dot{\tilde{m}}\left(t\right)=-S\left(\omega \left(t\right)\right)\left(\tilde{m}\left(t\right)-b_{m}\left(t\right)\right)=-S\left(\omega \left(t\right)\right)\tilde{m}\left(t\right)+S\left(\omega \left(t\right)\right)b_{m}\left(t\right).$$

Equation (17) can be rearranged to form the state transition Equation (18) for the KF application to estimate the $b_{m}\left(t\right)$ bias along with measurement $\tilde{m}\left(t\right)$:

(18) $$\left(\begin{array}{c} \dot{\tilde{m}}\left(t\right)\\ {\dot{b}}_{m}\left(t\right) \end{array}\right)=\left(\begin{array}{cc} -S(\omega (t)) & S(\omega (t))\\ 0 & 0 \end{array}\right)\left(\begin{array}{c} \tilde{m}\left(t\right)\\ b_{m}\left(t\right) \end{array}\right).$$

The measurement $\tilde{m}\left(t\right)$ is obtained from ${\left(\tilde{m}\left(t\right)\;\;b_{m}\left(t\right)\right)}^{T}$ as Equation (19):

(19) $$\tilde{m}\left(t\right)=\left(\begin{array}{cc} I & 0 \end{array}\right)\left(\begin{array}{c} \tilde{m}\left(t\right)\\ b_{m}\left(t\right) \end{array}\right).$$

In the derivation of Equation (18), it is again assumed that bias $b_{m}\left(t\right)$ does not change substantially with time, as assumed in the derivation of Equation (15). Equations (18) and (19) correspond to the process and measurement model in the application of the KF to the estimation of the bias $b_{m}\left(t\right)$ [24]. Applying the typical KF procedure using the models represented by Equations (18) and (19) provides an estimate of the bias in the magnetic field measurement [23]. The bias calibrated magnetic field expressed by Equation (20) is used in the remainder of the attitude estimation procedure:

(20) $$m\left(t\right)=\tilde{m}\left(t\right)-b_{m}\left(t\right),$$

where $b_{m}\left(t\right)$ is provided by the KF, which is implemented using Equations (18) and (19).

### 3.3. Measurement Model and Linearization of the Measurement Model

The proposed method uses the measured magnetic field and acceleration for the correction of the predicted state ${\hat{x}}^{-}\left(t_{k}\right)$, and the state covariance $P^{-}\left(t_{k}\right)$. The measurement model is the function which maps the state onto the magnetic field and acceleration measurements. The measurement model is nonlinear and needs to be linearized to apply the EKF approach.

First, the measurement model function h(x(t)) is derived. For the attitude estimation, the normalized measurement $z^{u}\left(t\right)$ is used as the measurement z(t):

(21) $$z\left(t\right)\equiv z^{u}\left(t\right)={\left(a^{u}\left(t\right),m^{u}\left(t\right)\right)}^{T}=h\left(x\left(t\right)\right)+w\left(t\right),\;w\left(t\right)∼N\left(0,R\left(t\right)\right).$$

The derivation of the measurement function for the acceleration is based on the assumption that the acceleration of the vehicle motion is negligible in comparison with gravity. The assumption ascertains that the normalized acceleration vector $a^{u}\left(t\right)={\left(a_{x}^{u}\left(t\right),a_{y}^{u}\left(t\right),a_{z}^{u}\left(t\right)\right)}^{T}$ corresponds to the unit vector in the upward direction, which is ${\left(0,0,-1\right)}^{T}$ in the NED coordinate frame. Therefore, the following Equation (22) describes the relationship between the unit acceleration vector and the attitude:

(22) $$a^{u}\left(t\right)=\left(\begin{array}{c} a_{x}^{u}\left(t\right)\\ a_{y}^{u}\left(t\right)\\ a_{z}^{u}\left(t\right) \end{array}\right)={}_{r}^{v}R\left(t\right)\left(\begin{array}{c} 0\\ 0\\ -1 \end{array}\right)={}_{v}^{r}R^{T}\left(t\right)\left(\begin{array}{c} 0\\ 0\\ -1 \end{array}\right)=\left(\begin{array}{c} S(\theta )\\ -S(\phi )C(\theta )\\ -C(\phi )C(\theta ). \end{array}\right)$$

Equation (22) describes the measurement model for the acceleration measurement.

The measurement model for the magnetic field measurement is based on the relationship between the geomagnetic field and the measured magnetic field. The measured magnetic field is the geomagnetic field in the sensor coordinate frame. The geomagnetic field vector $m_{g}$ consists of the northerly intensity $m_{g,x}$, easterly intensity $m_{g,y}$, and vertical intensity $m_{g,z}$ (positive downwards) of the geomagnetic field at a certain location on Earth:

(23) $$m_{g}={\left(m_{g,x},m_{g,y},m_{g,z}\right)}^{T}.$$

The geomagnetic field value depends on the location and altitude on Earth. In this paper, the latest model of the world magnetic model for 2015–2020 is used [25]. The magnetic field measurement m(t) is determined by applying rotation to the geomagnetic field $m_{g}$, as expressed by Equation (24):

(24) $$m\left(t\right)=\left(\begin{array}{c} m_{x}\left(t\right)\\ m_{y}\left(t\right)\\ m_{z}\left(t\right) \end{array}\right)={}_{r}^{v}R\left(t\right)\left(\begin{array}{c} m_{g,x}\\ m_{g,y}\\ m_{g,z} \end{array}\right)={}_{v}^{r}R^{T}\left(t\right)\left(\begin{array}{c} m_{g,x}\\ m_{g,y}\\ m_{g,z} \end{array}\right)=\left(\begin{array}{c} {}_{v}^{r}R_{x}^{T}\left(t\right)m_{g}\\ {}_{v}^{r}R_{y}^{T}\left(t\right)m_{g}\\ {}_{v}^{r}R_{z}^{T}\left(t\right)m_{g} \end{array}\right).$$

The magnetic field m(t) of Equation (24) is normalized to $m^{u}\left(t\right)$ to constitute the measurement vector z, as expressed by the following Equation (25):

(25) $$m^{u}\left(t\right)=\left(\begin{array}{c} m_{x}^{u}\left(t\right)\\ m_{y}^{u}\left(t\right)\\ m_{z}^{u}\left(t\right) \end{array}\right)=\frac{1}{∥m_{g}∥}\left(\begin{array}{c} {}_{v}^{r}R_{x}^{T}m_{g}\\ {}_{v}^{r}R_{y}^{T}m_{g}\\ {}_{v}^{r}R_{z}^{T}m_{g} \end{array}\right)=\left(\begin{array}{c} {}_{v}^{r}R_{x}^{T}m_{g}^{u}\\ {}_{v}^{r}R_{y}^{T}m_{g}^{u}\\ {}_{v}^{r}R_{z}^{T}m_{g}^{u} \end{array}\right),\;\;\;\mathrm{where}\;\;m_{g}^{u}={\left(m_{g,x}^{u},\;m_{g,y}^{u},\;m_{g,z}^{u}\right)}^{T}=\frac{m_{g}}{∥m_{g}∥}=\frac{m_{g}}{\sqrt{{m_{g,x}}^{2}+{m_{g,y}}^{2}+{m_{g,z}}^{2}}}.$$

In Equation (25), $m_{g}$ is normalized to $m_{g}^{u}$. Using Equation (13), $m_{x}^{u}\left(t\right)$, $m_{y}^{u}\left(t\right)$, and $m_{z}^{u}\left(t\right)$ are derived as follows:

(26) $$m_{x}^{u}\left(t\right)={}_{v}^{r}R_{x}^{T}\left(t\right)m_{g}^{u}=C\left(\theta \right)C\left(\psi \right)m_{g,x}^{u}+C\left(\theta \right)S\left(\psi \right)m_{g,y}^{u}-S\left(\theta \right)m_{g,z}^{u},$$

(27) $$\begin{array}{ll} m_{y}^{u}\left(t\right)\;=\;{}_{v}^{r}R_{y}^{T}\left(t\right)m_{g}^{u} & \;=\;\left\{S\left(\phi \right)S\left(\theta \right)C\left(\psi \right)-C\left(\phi \right)S\left(\psi \right)\right\}m_{g,x}^{u}\\ & +\left\{S\left(\phi \right)S\left(\theta \right)S\left(\psi \right)+C\left(\phi \right)C\left(\psi \right)\right\}m_{g,y}^{u}+S\left(\phi \right)C\left(\theta \right)m_{g,z}^{u}, \end{array}$$

(28) $$\begin{array}{ll} m_{z}^{u}\left(t\right)={}_{v}^{r}R_{z}^{T}\left(t\right)m_{g}^{u} & \;=\;\left\{C\left(\phi \right)S\left(\theta \right)C\left(\psi \right)+S\left(\phi \right)S\left(\psi \right)\right\}m_{g,x}^{u}\\ & +\left\{C\left(\phi \right)S\left(\theta \right)S\left(\psi \right)-S\left(\phi \right)C\left(\psi \right)\right\}m_{g,y}^{u}+C\left(\phi \right)C\left(\theta \right)m_{g,z}^{u}. \end{array}$$

Using Equations (22) and (25), the measurement model is derived as the function h(x(t)) expressed by Equation (29):

(29) $$z\left(t\right)=\left(\begin{array}{c} a_{x}^{u}\left(t\right)\\ a_{y}^{u}\left(t\right)\\ a_{z}^{u}\left(t\right)\\ m_{x}^{u}\left(t\right)\\ m_{y}^{u}\left(t\right)\\ m_{z}^{u}\left(t\right) \end{array}\right)=h\left(x\left(t\right)\right)=\left(\begin{array}{c} S(\theta )\\ -S(\phi )C(\theta )\\ -C(\phi )C(\theta )\\ {}_{v}^{r}R_{x}^{T}m_{g}^{u}\\ {}_{v}^{r}R_{y}^{T}m_{g}^{u}\\ {}_{v}^{r}R_{z}^{T}m_{g}^{u} \end{array}\right).$$

The linearized measurement model $H(t_{k})$ is derived from Equations (26)–(29):

(30) $$\begin{array}{cc} H(t_{k}) & \equiv {\left.\frac{\partial h(x)}{\partial x}\right|}_{x={\hat{x}}^{-}\left(t_{k}\right)}={\left.\left(\begin{array}{ccc} \frac{\partial a_{x}^{u}}{\partial \phi } & \frac{\partial a_{x}^{u}}{\partial \theta } & \frac{\partial a_{x}^{u}}{\partial \psi }\\ \frac{\partial a_{y}^{u}}{\partial \phi } & \frac{\partial a_{y}^{u}}{\partial \theta } & \frac{\partial a_{y}^{u}}{\partial \psi }\\ \frac{\partial a_{z}^{u}}{\partial \phi } & \frac{\partial a_{z}^{u}}{\partial \theta } & \frac{\partial a_{z}^{u}}{\partial \psi }\\ \frac{\partial m_{x}^{u}}{\partial \phi } & \frac{\partial m_{x}^{u}}{\partial \theta } & \frac{\partial m_{x}^{u}}{\partial \psi }\\ \frac{\partial m_{y}^{u}}{\partial \phi } & \frac{\partial m_{y}^{u}}{\partial \theta } & \frac{\partial m_{y}^{u}}{\partial \psi }\\ \frac{\partial m_{z}^{u}}{\partial \phi } & \frac{\partial m_{z}^{u}}{\partial \theta } & \frac{\partial m_{z}^{u}}{\partial \psi } \end{array}\right)\right|}_{x={\hat{x}}^{-}\left(t_{k}\right)} \end{array}.$$

In Equation (30), the time index $\left(t_{k}\right)$ is deleted for notational efficiency. The partial derivative elements of matrix $H(t_{k})$ are derived as follows:

(31) $$\begin{array}{lllllllll} \frac{\partial a_{x}^{u}}{\partial \phi } & = & 0 & \frac{\partial a_{x}^{u}}{\partial \theta } & = & C(\theta ) & \frac{\partial a_{x}^{u}}{\partial \psi } & = & 0, \end{array}$$

(32) $$\begin{array}{lllllllll} \frac{\partial a_{y}^{u}}{\partial \phi } & = & -C(\phi )C(\theta )\;\;\;\;\;\; & \frac{\partial a_{y}^{u}}{\partial \theta } & = & S(\phi )S(\theta )\;\;\;\;\;\; & \frac{\partial a_{y}^{u}}{\partial \psi } & = & 0, \end{array}$$

(33) $$\begin{array}{lllllllll} \frac{\partial a_{z}^{u}}{\partial \phi } & = & S(\phi )C(\theta ) & \frac{\partial a_{z}^{u}}{\partial \theta } & = & C(\phi )S(\theta ) & \frac{\partial a_{z}^{u}}{\partial \psi } & = & 0, \end{array}$$

(34) $$\frac{\partial m_{x}^{u}}{\partial \phi }=0,$$

(35) $$\frac{\partial m_{x}^{u}}{\partial \theta }=-S\left(\theta \right)C\left(\psi \right)m_{g,x}^{u}-S\left(\theta \right)S\left(\psi \right)m_{g,y}^{u}-C\left(\theta \right)m_{g,z}^{u},$$

(36) $$\frac{\partial m_{x}^{u}}{\partial \psi }=-C\left(\theta \right)S\left(\psi \right)m_{g,x}^{u}+C\left(\theta \right)C\left(\psi \right)m_{g,y}^{u},$$

(37) $$\begin{array}{ll} \frac{\partial m_{y}^{u}}{\partial \phi }= & \left\{C\left(\phi \right)S\left(\theta \right)C\left(\psi \right)+S\left(\phi \right)S\left(\psi \right)\right\}m_{g,x}^{u}+\left\{C\left(\phi \right)S\left(\theta \right)S\left(\psi \right)-S\left(\phi \right)C\left(\psi \right)\right\}m_{g,y}^{u},\\ & +C\left(\phi \right)C\left(\theta \right)m_{g,z}^{u}, \end{array}$$

(38) $$\frac{\partial m_{y}^{u}}{\partial \theta }=S\left(\phi \right)C\left(\theta \right)C\left(\psi \right)m_{g,x}^{u}+S\left(\phi \right)C\left(\theta \right)S\left(\psi \right)m_{g,y}^{u}-S\left(\phi \right)S\left(\theta \right)m_{g,z}^{u},$$

(39) $$\frac{\partial m_{y}^{u}}{\partial \psi }=-\left\{S\left(\phi \right)S\left(\theta \right)S\left(\psi \right)+C\left(\phi \right)C\left(\psi \right)\right\}m_{g,x}^{u}+\left\{S\left(\phi \right)S\left(\theta \right)C\left(\psi \right)-C\left(\phi \right)S\left(\psi \right)\right\}m_{g,y}^{u},$$

(40) $$\begin{array}{ll} \frac{\partial m_{z}^{u}}{\partial \phi }= & \left\{-S\left(\phi \right)S\left(\theta \right)C\left(\psi \right)+C\left(\phi \right)S\left(\psi \right)\right\}m_{g,x}^{u}-\left\{S\left(\phi \right)S\left(\theta \right)S\left(\psi \right)+C\left(\phi \right)C\left(\psi \right)\right\}m_{g,y}^{u},\\ & -S\left(\phi \right)C\left(\theta \right)m_{g,z}^{u}, \end{array}$$

(41) $$\frac{\partial m_{z}^{u}}{\partial \theta }=C\left(\phi \right)C\left(\theta \right)C\left(\psi \right)m_{g,x}^{u}+C\left(\phi \right)C\left(\theta \right)S\left(\psi \right)m_{g,y}^{u}-C\left(\phi \right)S\left(\theta \right)m_{g,z}^{u},$$

(42) $$\frac{\partial m_{z}^{u}}{\partial \psi }=\left\{-C\left(\phi \right)S\left(\theta \right)S\left(\psi \right)+S\left(\phi \right)C\left(\psi \right)\right\}m_{g,x}^{u}+\left\{C\left(\phi \right)S\left(\theta \right)C\left(\psi \right)+S\left(\phi \right)S\left(\psi \right)\right\}m_{g,y}^{u}.$$

The linearized matrix $H(t_{k})$ is used in the correction procedure described in Section 3.4.

### 3.4. Correction of Predicted State and State Covariance

The state representing the attitude is finally estimated as $\hat{x}\left(t_{k}\right)$ by adjusting the predicted state ${\hat{x}}^{-}\left(t_{k}\right)$ as expressed by the following Equation (43):

(43) $$\hat{x}\left(t_{k}\right)={\hat{x}}^{-}\left(t_{k}\right)+K\left(t_{k}\right)\left\{z\left(t_{k}\right)-h\left({\hat{x}}^{-}\left(t_{k}\right)\right)\right\}.$$

The Kalman gain $K(t_{k})$ in Equation (43) is determined from the linearized measurement model $H(t_{k})$ and the predicted covariance matrix $P^{-}\left(t_{k}\right)$:

(44) $$K\left(t_{k}\right)=P^{-}\left(t_{k}\right)H{\left(t_{k}\right)}^{T}{\left\{H\left(t_{k}\right)P^{-}\left(t_{k}\right)H{\left(t_{k}\right)}^{T}+R\left(t_{k}\right)\right\}}^{-1}.$$

The predicted covariance $P^{-}\left(t_{k}\right)$ is also corrected to $P(t_{k})$ by Equation (45):

(45) $$P\left(t_{k}\right)=P^{-}\left(t_{k}\right)-K\left(t_{k}\right)H\left(t_{k}\right)P^{-}\left(t_{k}\right).$$

Equations (43) and (45) complete the attitude estimation procedure. The estimated state $\hat{x}\left(t_{k}\right)$ and covariance $P(t_{k})$ of Equations (43) and (45) are used in the next iteration of the EKF procedure to predict ${\hat{x}}^{-}\left(t_{k+1}\right)$ and $P^{-}\left(t_{k+1}\right)$ using Equations (9) and (10).

### 3.5. Use of Horizontal Component of Magnetic Field Measurement

In the proposed field measurement approach, the magnetic field measurement affects the roll and pitch estimation, and the yaw estimation. The acceleration measurement plays a major role in estimating the roll and pitch, and the estimated roll and pitch have enough accuracy and stability for use in underwater navigation, where the acceleration of vehicle motion is negligible in comparison with gravity. Because the magnetic field measurement is more vulnerable to environmental distortion than the acceleration measurement, the use of magnetic field measurement occasionally deteriorates the estimation of roll and pitch if the magnetic field measurement is not effectively calibrated to reduce the distortion effect. This happens when the range of the roll and pitch motion is limited. Thus, the estimation of the vertical bias component is not sufficiently accurate to calibrate the magnetic field measurement.

In this paper, we propose that only the horizontal component of the magnetic field measurement should be used to prevent the adverse effect of the magnetic field measurement on the estimation of the roll and pitch, when the magnetic field is significantly distorted or the vertical bias component cannot be calibrated, owing to the limited roll and pitch motion.

The horizontal component of the magnetic field is the magnetic field projected onto the North–East plane (NE plane) in the NED coordinate frame. The proposed method extracts the horizontal component ${}^{h}m\left(t\right)$ of the magnetic field measurement from the raw magnetic field measurement m(t) by subtracting the vertical component of the magnetic field using Equation (46):

(46) $${}^{h}m\left(t\right)=m\left(t\right)-\frac{m(t)\cdot a(t)}{a(t)\cdot a(t)}a\left(t\right).$$

The last term of Equation (46) represents the projection of the magnetic field m(t) onto vector a(t), which is assumed to be perpendicular to the NE plane, as assumed when the roll and pitch are calculated from the acceleration measurement. The horizontal component ${}^{h}m\left(t\right)$ of the magnetic field measurement m(t) is normalized to ${}^{h}m^{u}\left(t\right)$, as expressed by Equation (47):

(47) $${}^{h}m^{u}\left(t\right)=\frac{{}^{h}m\left(t\right)}{∥{}^{h}m\left(t\right)∥}={\left({}^{h}m_{x}^{u}\left(t\right),{}^{h}m_{y}^{u}\left(t\right),{}^{h}m_{z}^{u}\left(t\right)\right)}^{T}.$$

In this approach, the measurement consists of the normalized acceleration and normalized horizontal magnetic field, as expressed by Equation (48):

(48) $$z\left(t\right)=z^{u}\left(t\right)={\left(a_{x}^{u}\left(t\right),a_{y}^{u}\left(t\right),a_{z}^{u}\left(t\right),{}^{h}m_{x}^{u}\left(t\right),{}^{h}m_{y}^{u}\left(t\right),{}^{h}m_{z}^{u}\left(t\right)\right)}^{T}.$$

Because the NE-plane magnetic field measurement is used in the measurement update procedure of the EKF, the NE-plane geomagnetic field ${}^{h}m_{g}$ should be used instead of the full geomagnetic field $m_{g}$, which is given by Equation (24). The NE-plane geomagnetic field ${}^{h}m_{g}$ is simply the NE component of $m_{g}$, as expressed by Equation (49):

(49) $${}^{h}m_{g}={\left(m_{g,x},m_{g,y},0\right)}^{T}.$$

The use of ${}^{h}m\left(t\right)$ and ${}^{h}m_{g}$ instead of m(t) and $m_{g}$ completes the approach where only the horizontal component of the magnetic field measurement is used to remove the adverse effect originating from the use of the full magnetic field measurement in the estimation of roll and pitch.

## 4. Experiments and Discussion

The proposed method was evaluated and compared with other methods through experiments inside two test pools with different remotely operated vehicles (ROVs) and sensors. The compared methods were NECF, SR, EKF, CF, and the method installed into the used AHRS by the manufacturer (LORD MicroStrain, Williston, VT, USA). The internal method is a complementary filter (CF) and provides the attitude in Euler angles [26]. In the two experiments, the attitude and location were represented in the NED coordinate frame. The origin of the NED coordinate frame for the location description is the initial location, where the UUV starts a predefined motion through a given trajectory for each test. The method proposed in this paper is called the FM method, which stands for field measurement method.

**Figure 1:**
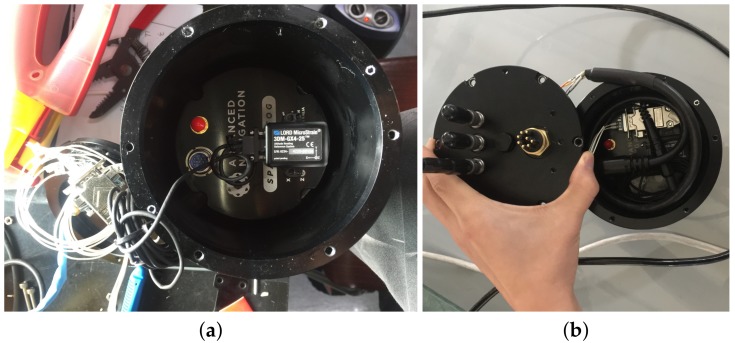
Installation of FOG and AHRS in experiment 1. (**a**) FOG and AHRS in the waterproof hull; (**b**) cover of the waterproof hull.

**Figure 2:**
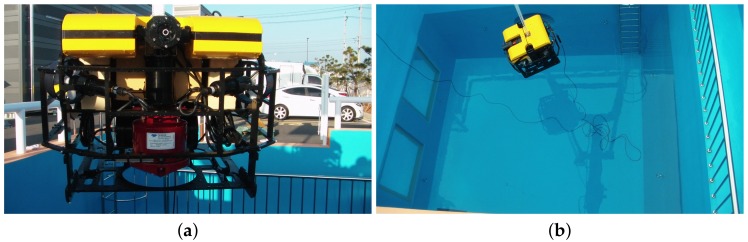
UUV and test tank used in experiment 1. (**a**) UUV front view; (**b**) launching UUV into test tank.

**Figure 3:**
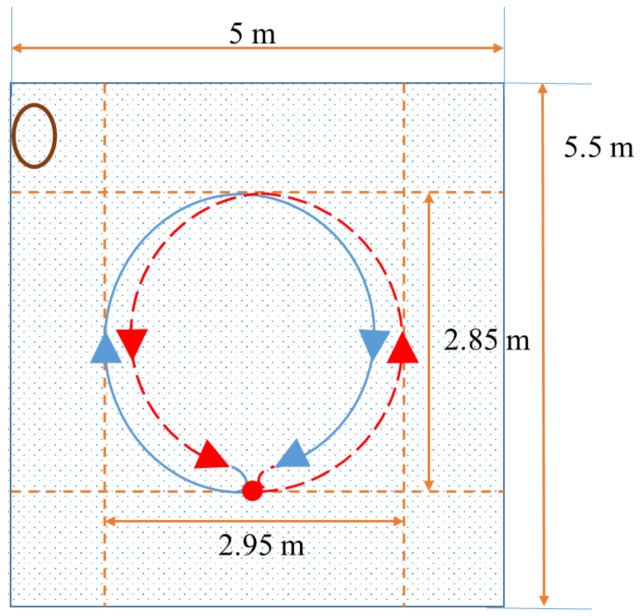
Experiment 1 trajectory shown in test pool.

The first experiment uses an AHRS (LORD Microstrain 3DM-GX4 25) [26], a fibre optic gyroscope (FOG, Advanced Navigation, Spatial Fog, Sydney, NSW, Australia) [27], and a DVL (Teledyne RD Instruments, Navigator Doppler Velocity Log, Poway, CA, USA) [28]. The attitude provided by the FOG is used as a reference with which the attitude estimated by the abovementioned methods are compared. Figure 1 shows the installation of the AHRS and FOG in water-proof housing. The performance of the FOG is shown in Table 1. Table 2 describes the performance of the AHRS. Figure 2 shows the UUV used for the experiment along with the test pool. The test pool is located in the city of Gwangju, Korea, and has a length and depth of 5.5 m, 5.0 m, respectively. According to the world magnetic model for 2015–2020 at the location of the test pool, the geomagnetic field was set to $m_{g}={\left(0.307198G,-0.041355G,0.390659G\right)}^{T}$ [25]. The UUV was controlled remotely to navigate through the predefined trajectories in the pool. The UUV had five degrees of freedom in motion with six thrusters: four horizontal thrusters for surge, sway, and yaw motion, and two vertical thrusters for roll and heave motion. Figure 3 shows the trajectory through which the UUV navigated within the test pool. The UUV moved in a circular trajectory from its initial location. Every time it reached back to its initial location at the end of one circular motion, it turned around in place to follow the reverse direction and trace back to the circular trajectory. The travel distance and time were 264 m, and 34 min and 20 s, with an average speed of 0.21 m/s when the UUV was moving, excluding the rotation at the end of each circulation.

**Figure 4:**
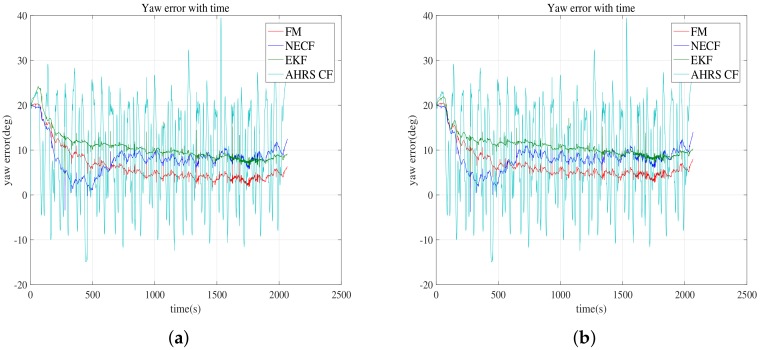
Error of estimated yaw with respect to the yaw provided by FOG is compared. (**a**) bias estimation is used; (**b**) bias estimation is not used.

**Figure 5:**
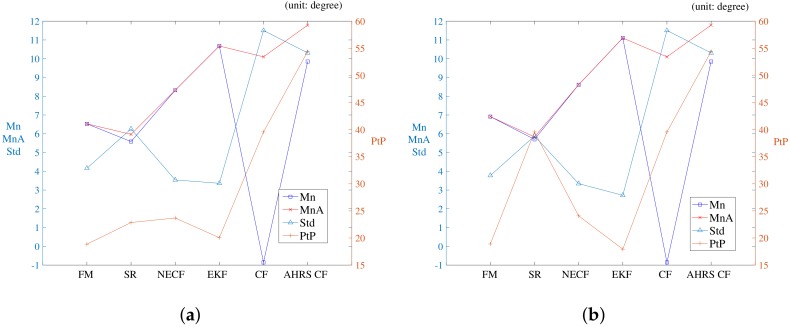
Average, average absolute error, standard deviation, and peak to peak of attitude error obtained with considered methods in experiment 1. (**a**) bias compensated; (**b**) bias not compensated.

Figure 4 compares the yaw estimation of the considered methods. The errors in the roll and pitch are not displayed because the errors of all considered methods are comparable. The reference to the error is the attitude provided by the FOG. For the attitude error, Table 3 shows the average (Mn), average absolute error (MnA), standard deviation (Std), and maximum peak to peak (PtP) of the error. In Table 3, the AHRS CF indicates the CF method implemented internally in the AHRS, while CF represents the CF implemented in this study. Figure 5 depicts the error statistics graphically to make the comparison easier. The mean, average absolute error, standard deviation, and peak to peak of the error are shown.

Although the roll and pitch estimation errors for all methods are comparable, a difference in the yaw estimation error is noticeable. Therefore, the discussion regarding the results is focused on the statistical comparison of the yaw estimation error. Figure 5 shows a graphical representation of the mean, average absolute error, standard deviation, and peak to peak of the error. As shown in Table 3 and Figure 5, the proposed FM method achieves the best performance in peak to peak, and the second best performance with regard to the mean and average absolute error. The standard deviation performance of the FM ranks third. Although the performance of SR in the mean error and average absolute error are better than the performance of the proposed method, the performance of the standard deviation and peak to peak of the error for SR are inferior in comparison with the same performance of the proposed FM method. In comparison with the other methods, it can be generally considered that the FM method outperforms the other methods.

**Figure 6:**
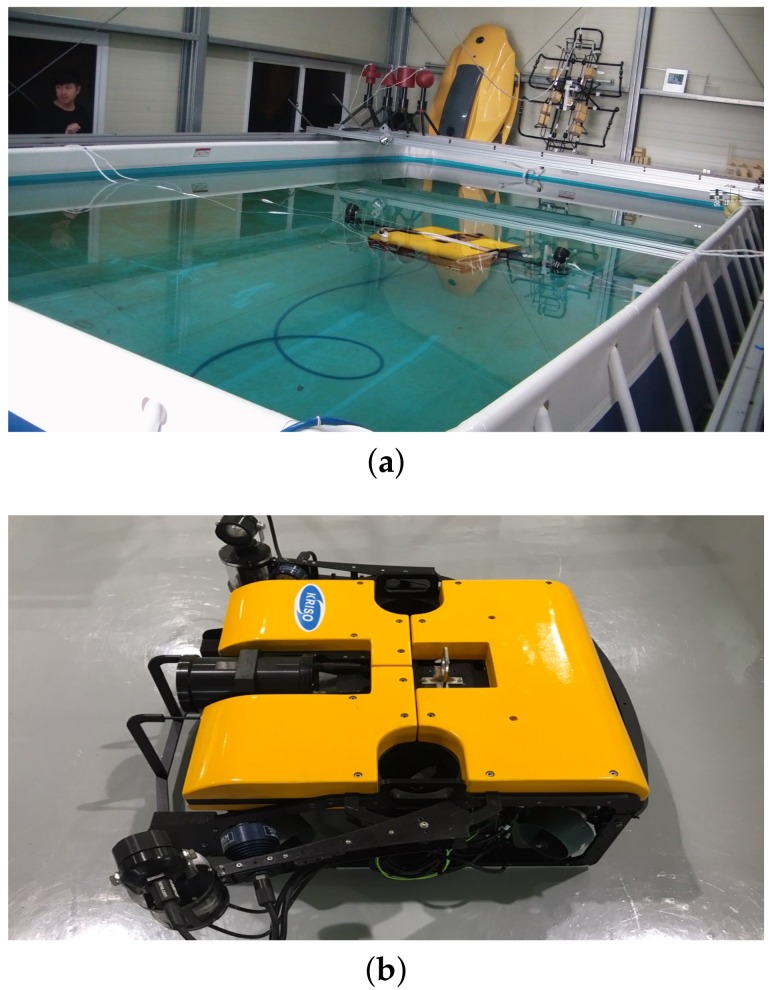
Test pool and UUV used in experiment 2. (**a**) test pool; (**b**) UUV.

**Figure 7:**
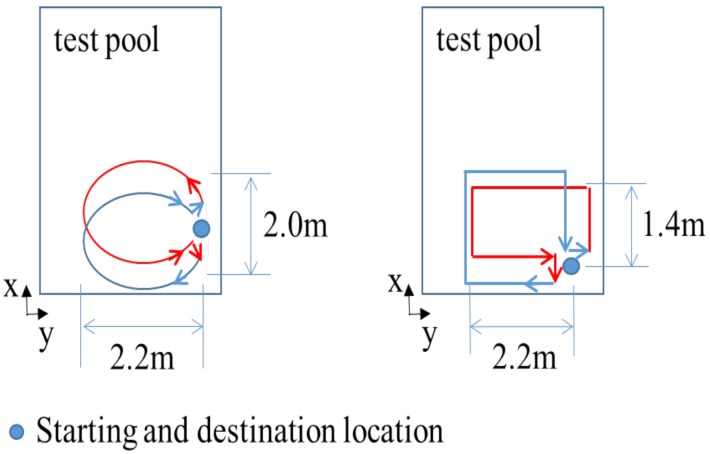
Two trajectories in navigation experiment 2 [22].

The second experiment used an AHRS (LORD Microstrain 3DM-GX3 25, LORD MicroStrain, Williston, VT, USA) [29] and a DVL (LinkQuest NavQuest 600 Micro, San Diego, CA, USA) [30] installed onto the UUV. The test pool was located in the city of Daejeon, Korea, and the geomagnetic field was set to $m_{g}={\left(0.300684G,-0.042526G,0.401987G\right)}^{T}$ [25], in accordance with the 2015–2020 world magnetic model with regard to the specific location. The test pool and the UUV are shown in Figure 6. The UUV navigated through the two different trajectories shown in Figure 7—namely, a circular trajectory and a rectangular trajectory. For each trajectory, once the robot circulated and reached its initial location, it turned back and traced the trajectory to the initial location by following the reverse direction. Then, it turned back and traced the previous path, and moved again toward the initial location. The robot repeated this back and forth navigation, and finally returned to its initial location such that the destination of the navigation was the robot’s initial location. The total travel distance of the circular motion was 282.7 m, and the total distance of the rectangular motion was 100.8 m [22]. Because the second experiment did not use a high-end attitude sensor, the attitude estimation performance was analyzed indirectly by comparing the accuracy of the dead-reckoned localization, which depends on the estimated attitude.

**Figure 8:**
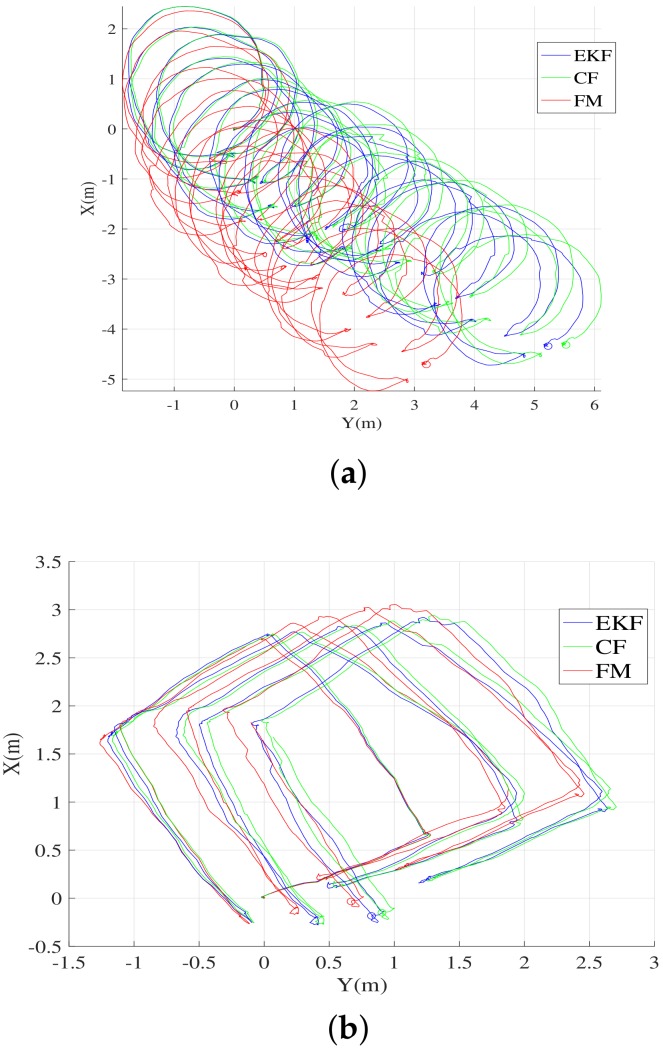
Trajectories calculated in experiment 2. (**a**) circular trajectory; (**b**) rectangular trajectory.

Figure 8 shows the trajectories dead-reckoned using the estimated attitude and velocity measured by the DVL. Because the DVL measures the velocity in the sensor coordinate frame, the velocity is transformed to the velocity in the NED coordinate frame by using the attitude estimated by the considered methods. The NED velocity was dead-reckoned to produce the trajectory in the NED coordinate frame. Therefore, the accuracy of the calculated trajectory in the NED coordinate frame partially indicates the performance of attitude estimation. The UUV began moving from the origin (0,0,0) of the coordinate system, and ended its motion at the same location after circulating the trajectory. The errors in the estimated attitude and measured velocity cause the calculated UUV location to drift with time. The drift of the calculated final destination from the true destination (0,0,0) is regarded as a measure of accuracy for the estimated attitude.

Table 4 describes the distance error of the estimated location at the final destination. Although the drift in the *z*-direction is not shown in Figure 8, the *z*-directional error adds to the distance error on the xy plane shown in Figure 8. Thus, it results in the distance error presented in Table 4. The distance error for the FM is shorter than the errors of the other methods. Although the distance error is not the exact measure of accuracy for the estimated attitude, the result proves that the calculated trajectory based on the proposed method is better than that of the other methods, and indirectly indicates that the proposed attitude estimation method is the most probable one amongst all methods compared in this study.

Reduction of computational expenses are critical for the practical use of the algorithm since the computational capability is constrained by the limitations in space, weight, power consumption, and price available within the underwater vehicles. Table 5 compares the computation time for the five methods. The table lists the time required for one iteration of each estimation algorithm. All the algorithms are tested using Matlab version R2018a (MathWorks Inc., Natick, MA, USA) under Windows 10 Pro(x64) system (Microsoft Corp., Redmond, WA, USA) on the desktop computer with CPU Intel Core i7-7700K (Intel Corp., Santa Clara, CA, USA), 4.20 GHz, 16 GB of memory.

Table 5 indicates that the proposed method takes the longest computation time. However, all the algorithms run in comparable computation time, which is less than 0.20 ms. The proposed method can run over 5.0 KHz, and it is enough for real-time implementation. The NECF and EKFs have been widely used for underwater navigation. As the computation time for NECF and EKFs are acceptable for practical underwater navigations, it is expected that the computation time for the proposed method is also allowable for real-time application. Memory usage is another important factor for implementation of the algorithms for practical use. For the Matlab implementation, the proposed method uses 3864 bytes of variables, which is considered reasonable because the other methods also require comparable amount of variables with the proposed method.

In order to use the method for navigation in open water environment, the following aspects should be taken into account besides the computation expenses: (1) periodic position and attitude fixing is needed; and (2) geomagnetic field reference should be updated as the vehicle navigates from one place to another. Underwater navigation which depends on DVL and inertia measurement unit (IMU) inevitably suffer from accumulation of error in location and heading as shown in Figure 8. Therefore, periodic correction of the location and attitude is required. In some applications, the vehicle periodically surfaces and corrects the location and heading using the GPS data. In some other cases where the vehicle navigates in a limited navigation space, acoustic baseline system such as USBL, short baseline (SBL), or long baseline (LBL) can be used for the correction.

The proposed method requires geomagnetic field reference data, which depends on geographic location of the underwater vehicle. Table 6 shows the geomagnetic field values of two locations which are 9.1 km apart from each other. The geomagnetic field values are for the date of 9 January 2019, which are calculated using the world magnetic model (WMM) 2015, version 2, updated in September 2018 [31]. The distance between the two places are calculated using the Universal Transverse Mercator (UTM) coordinates with world geodetic system (WGS)-84 ellipsoid data. Table 6 indicates that the variation of geomagnetic field values is small enough to be ignored for application of the proposed method when the locations are within 9.1 km range.

The geomagnetic field could be considered constant if the vehicle’s workspace is within several kilometers of bound. However, if the vehicle navigates around significantly large range, the geomagnetic field should be updated. For the update, the geomagnetic model should be installed into the vehicle control processor system. Alternatively, the geomagnetic field value can be updated through wireless communication when the vehicle surfaces. Because the estimation error of location by the proposed method accumulates with time and travel distance, the geomagnetic field calculated based on the estimated location also deviates from the true value. Therefore, periodic fix of location is needed using either GPS reception at the surfacing period or using acoustic baseline systems.

## 5. Conclusions

This paper proposes a method of estimating the attitude of an underwater robot using measurement by an AHRS. The proposed method uses a magnetic field and acceleration measurement at the correction stage of the filtering. Unlike existing EKF based methods, the proposed method uses the field measurement as is, by directly comparing the measured field with the known field strength specific to the geographic locations where the UUV operates. The proposed method was compared with five other methods using measurement data sampled through two experiments in test tanks. The statistical analysis for the attitude error revealed that the proposed method performed better than, or at least comparably, with the other considered methods.

The proposed formulation uses Euler angles for the state transition and measurement model. It is expected that the use of an alternative representation for the attitude, such as a quaternion or rotation matrix, will result in a different state transition model and measurement model. In future work, an invariant observer which uses field measurement will be adopted [20] to further develop this study. It is thought that the incorporation of the field measurement approach with an alternative attitude representation utilizing the invariance property could improve the convergence performance and accuracy of attitude estimation.

## Figures and Tables

**Table 1:** Specification of fibre optic gyroscope (FOG) used in experiment 1 to provide reference attitude for comparison.

Parameter Value	
Roll and pitch accuracy	0.01${}^{\circ }$
Heading accuracy (global navigation satellite system (GNSS) aided)	0.05${}^{\circ }$
Heading accuracy (north seeking only)	0.25${}^{\circ }$ secant latitude
Output data rate	Up to 1000 Hz

**Table 2:** Specification of AHRS used in experiment 1.

Parameter	Value
Gyroscope bias instability	10${}^{\circ }/$h
Accuracy of CF attitude estimation (static)	$\pm 0.5^{\circ }$
Accuracy of CF attitude estimation (dynamic)	$\pm 2.0^{\circ }$

**Table 3:** Average of error, average of absolute error, standard deviation, and peak to peak of attitude estimation error in experiment 1 (dimension in degrees).

Method	Bias Compensation		Mn (unit: deg)				MnA (Unit: deg)		
			Roll	Pitch	Yaw		Roll	Pitch	yaw
**FM**	compensated		−0.5693	0.1239	6.5286		0.5709	0.2604	6.5286
	not compensated		−0.5692	0.1238	6.9146		0.5709	0.2604	6.9146
**SR**	compensated		−0.8603	0.1497	5.5848		0.8615	0.6181	5.9690
	not compensated		−0.8692	0.1658	5.7003		0.8701	0.5965	5.8336
**NECF**	compensated		−0.3960	0.0924	8.3195		0.3961	0.1682	8.3200
	not compensated		−0.3951	0.0849	8.6126		0.3952	0.1600	8.6132
**EKF**	compensated		−0.7011	0.1697	11.1049		0.7075	0.2772	10.6770
	not compensated		−0.7011	0.1697	11.1049		0.7075	0.2772	11.1049
**CF**	compensated		−0.4251	0.0749	−0.8583		0.4857	0.2551	10.1000
	not compensated		−0.4251	0.0749	−0.8583		0.4857	0.2551	10.1000
**AHRS CF**	not known		−0.6779	0.1340	9.8494		0.6825	0.2670	11.7897
			**Std (unit: deg)**				**PtP (Unit: deg)**		
			**Roll**	**Pitch**	**Yaw**		**Roll**	**Pitch**	**Yaw**
**FM**	compensated		0.3426	0.2857	4.1599		1.4407	1.2710	18.8553
	not compensated		0.3431	0.2862	3.7747		1.4422	1.2721	18.8933
**SR**	compensated		0.4425	0.7037	6.2573		2.1397	3.3442	22.8592
	not compensated		0.3511	0.6671	5.8768		2.0830	3.2952	22.1971
**NECF**	compensated		0.1891	0.1829	3.5384		0.9844	1.5147	23.6874
	not compensated		0.2056	0.1741	3.3417		1.0316	1.4915	24.0799
**EKF**	compensated		0.3658	0.3072	3.3592		1.7996	1.4153	20.0542
	not compensated		0.3658	0.3072	2.7169		1.7996	1.4153	17.9347
**CF**	compensated		0.3952	0.3016	11.5041		1.9221	1.4122	39.5673
	not compensated		0.3952	0.3016	11.5038		1.9221	1.4122	39.5673
**AHRS CF**	not known		0.3588	0.3047	10.3116		1.5245	1.3126	54.4047

**Mn:** Average error; **MnA:** Average absolute error; **Std:** Standard deviation of error; **PtP:** Peak to peak of error.

**Table 4:** Calculated location and distance error at destination. The location was dead-reckoned based on the attitude estimated by the considered methods.

Method	Calculated Location (Unit: m)		Distance Error (Unit: m)	
	Circular Traj.	Rectangular Traj.	Circular Traj.	Rectangular traj.
**FM**	(−4.7123, 3.2019, −1.8038)	(−0.0358, 0.6696, −0.7202)	5.9759	0.9840
**EKF**	(−4.3445, 5.2280, −1.7294)	(−0.1855, 0.8266, −0.6372)	7.0141	1.0600
**CF**	(−4.3212, 5.5269, −2.1790)	(−0.1524, 0.9068, −0.7710)	7.3462	1.2000

**Table 5:** Computation time for the considered methods.

Method	FM	SR	NECF	EKF	CF
**Computation Time** (unit: ms)	0.165	0.156	0.142	0.151	0.135

**Table 6:** Comparison of geomagnetic field of two locations 9.1 km apart, for the date of 9 January 2019.

	Latitude (deg.)	Longitude (deg.)	Altitude (m)	Geomagnetic Field (Unit: nT)		
				X (North)	Y (East)	Z (Down)
**Location 1**	35.0	130.0	0.0	30,776	−4306	37,892
**Location 2**	35.0	130.1	0.0	30,773	−4307	37,859
